# Thermosensitive Micelles Gel to Deliver Quercetin Locally for Enhanced Antibreast Cancer Efficacy: An In Vitro Evaluation

**DOI:** 10.1049/2023/7971492

**Published:** 2023-11-02

**Authors:** Yanxue Sun, Yun Bai, Silu Liu, Shuxia Cui, Pengcheng Xu

**Affiliations:** ^1^Department of Pharmaceutical Engineering, College of Pharmacy, Inner Mongolia Medical University, Hohhot, China; ^2^Department of Anesthesiology, The Second Affiliated Hospital of Inner Mongolia Medical University, Hohhot, China

## Abstract

Although quercetin is low cytotoxicity to normal human cells, quercetin is effective against the growth of some tumors. Given the poor blood stability in vivo, insolubility, low delivery efficiency, and poor medicinal properties of quercetin, we developed a local drug delivery system comprising quercetin core's polymer micelles and F127 hydrogel stroma. In vitro evaluation revealed that quercetin core's polymer micelles have excellent antitumor activity and could inhibit the multiplication of 4T1 breast cancer cells through the apoptosis pathway. Meanwhile, a rheological study revealed that the quercetin core's micelles gel possessed excellent properties of hydrogel formation and injectability of liquid preparation as a local drug delivery system after the quercetin core's polymer micelles were loaded into the F127 hydrogel stroma. Our study findings indicated that the drug stability and stable release capacity of quercetin were vastly improved with the composite formulation of the micelles gel. This not only realized drug injectability but also drug storage in the semisolid form, which is a more comfortable and slower drug-releasing form that will eventually exert a proper therapeutic effect. In conclusion, quercetin micellar hydrogel system has better antitumor activity and excellent hydrogel properties.

## 1. Introduction

Cancer is a life-threatening disease and has affected the world for a long time [1]. There are different types of cancer treatment methods; however, with the currently emerging treatment regimens, immunotherapy and cytotherapy are gaining popularity. However, both these methods have limitations. In brief, immunotherapy targets a limited cell population owing to poor responsiveness [2]. Although cytotherapy has specifically been recognized as an effective treatment for hematologic malignancies, it is forced into a great frustrated helpless in treating solid tumors due to poor tumor migration, infiltration of physical barriers, and active tumor suppression [3]. Currently, chemotherapy is regarded as the main treatment modality for cancer [4]; however, the lack of tumor targeting and the associated severe toxicity effects hinder the further development of chemotherapy [5]. Although small molecular targeted drugs have improved the targeting ability of chemotherapy, the accompanying skin toxicity effects limit the treatment potential [6]. Therefore, it is extremely urgent to develop an efficient high spatiotemporal drug delivery system for improving the precision and targeting of chemotherapeutic agents.

A topical drug delivery system through intratumoral and peritumoral injections with a more accurate dosage can enhance the therapeutic index for superficial tumors, thereby reducing the associated systemic adverse effects [7]. For example, for skin, pancreatic, and breast cancers, treatment via the local administration of chemotherapeutic agents to targeted sites demands extra care [8–10]. Local drug delivery systems, which have far-reaching significance, can adjust the drug targeting or dosages to ensure the efficacy of the chemotherapeutic agent and lower the associated systemic toxicity. Injectable thermosensitive hydrogels, e.g., F127, which possess good biocompatibility and function at lower critical solution temperatures, can also be used. That is, such hydrogels can be free-flowing at lower temperatures and immediately transmute into the solid state subject to changes in the physiological temperature and can achieve local drug accumulation that can release at the tumor site and effectively improve the drug concentration at the target site.

The discovery of new drugs from natural products is still an important research topic, and some of these drugs are often developed as anticancer drugs. Paclitaxel is an excellent example, which was developed and marketed as liposomes, micelles, albumin, etc. [11, 12]. Inspired by this successful case, we decided to use natural quercetin, which has selective cytotoxicity and exhibits weak cytotoxicity to normal human cells in contrast with cancer cells [13–15], which embodies its unique superiority among all chemotherapeutics agents. However, quercetin is insoluble and has a striking shortcoming: it is easily oxidized and unstable in vivo, which makes its use in drug development and clinical settings challenging [16, 17]. In this study, we showed that the polymer micelle system achieved effective encapsulation of quercetin into the hydrophobic polymer core to improve the shortcomings associated with solubility and delivery efficiency caused by exposure to surroundings, thereby formulating the payload of the quercetin core's polymer micelles in an excellent F127 hydrogel fellow, realizing sustained and stable localized drug release. Furthermore, we performed assessments related to drug particles, morphology, rheological behavior, drug release and retention in vitro, cytotoxicity, and apoptosis in terms of the in vitro antitumor efficacy of 4T1 breast cancer cells.

## 2. Materials and Methods

### 2.1. Materials

Quercetin was purchased from Sinopharm Chemical Reagent Co., Ltd. (Shanghai, China). Distearoyl phosphoethanolamine–polyethylene glycol_2000_ (DSPE–PEG_2000_) was purchased from NOF Corporation (Tokyo, Japan). Coumarin 6 was purchased from Shanghai Maokang Biotechnology Co., Ltd. (Shanghai, China). Pluronic® F127 was purchased from Shanghai Aladdin Biochemical Technology Co., Ltd. (Shanghai, China). 3-(4,5-Dimethylthiazolyl-2)-2,5-diphenyltetrazolium bromide (MTT) was purchased from Beijing Coolaber Technology Co., Ltd. (Beijing, China). The annexin V-fluorescein isothiocyanate/propidium iodide (Annexin V-FITC/PI) kit was obtained from BioSharp Biotechnology Co., Ltd. (Anhui, China).

### 2.2. Cells

4T1 breast cancer cells were obtained from the Institute of Basic Medical Sciences, the Chinese Academy of Medical Science (Beijing, China).

### 2.3. Experimental Methods

#### 2.3.1. Preparation of Qu@PM, Qu@PM@Gel, C6@PM@Gel, and Free C6@Gel

The thin-film hydration method was used to prepare quercetin polymer micelles (Qu@PM). Briefly, quercetin and DSPE–PEG_2000_ were weighed separately and codissolved in a mixture of methanol and dichloromethane in an eggplant-shaped bottle. The solvents were then evaporated by a rotary evaporator at 37°C until a dry film was formed. The prepared film was quickly hydrated in ultrapure water to obtain the micellar solution. Qu@PM@Gel was formulated through the following methods. F127 (0.5 g) was dissolved in 1 mL of Qu@PM solution, and the mixture was stirred at 4°C till a clear solution was obtained. A blank hydrogel was prepared by stirring F127 granules (0.5 g) in 1 mL of ultrapure water at 4°C.

The C6 polymer micelles gel (C6@PM@Gel) was prepared using the same procedure. Free C6@Gel was prepared by adding C6, which was solubilized using dimethyl sulfoxide to F127 granules in an aqueous solution. All of the procedures were conducted in the dark.

#### 2.3.2. Characterization of Qu@PM and the Qu@PM@Gel

The particle size and zeta potential of Qu@PM were determined using the Zetasizer Nano ZS (Malvern Instrument) at 25°C. The morphology of the Qu@PM and Qu@PM@Gel were investigated using transmission electron microscopy (TEM) and scanning electron microscopy (SEM), respectively. The unencapsulated drug was removed by centrifugal method, and the content of quercetin was measured by ultraviolet spectrophotometry. The encapsulation efficiency (EE) and drug loading (DL) were calculated using Equations (1) and (2), respectively.

(1) $$\begin{array}{c} EE\%=\left(\frac{W_{a}}{W_{b}}\right)\times 100\%, \end{array}$$

(2) $$\begin{array}{c} DL\%=\left(\frac{W_{a}}{W_{c}}\right)\times 100\%, \end{array}$$

where *W*_a_, *W*_b_, and *W*_c_ represented the content of the encapsulated drug, the total content of quercetin, and the total content of micellar particles, respectively.

#### 2.3.3. Rheological Analysis

The rheological behaviors of the blank hydrogel and the Qu@PM@Gel were studied using a rheometer (T.A. Instrument, USA), and hydrogel discs with a 20 mm diameter and 1,000 *μ*m height were used. The viscosity of the blank hydrogel and Qu-PM-Gel with changes in the shear rate was measured at 25°C. A temperature sweep test was performed to determine the gel formulation temperature, ranging from 4 to 40°C. The storage modulus (*G*′) and loss modulus (*G*″) of the blank hydrogel and the Qu@PM@Gel were measured, with changes in the frequency ranging from 0 to 100 Hz. With the amplitude ranging from 0% to 100%, a strain sweep test was conducted to determine the linear viscoelastic region at 37°C.

#### 2.3.4. In Vitro Release Behaviors of Free C6, C6@PM, C6@PM@Gel, and Free C6@Gel

The in vitro release of the free C6, C6@PM, C6@PM@Gel, and free C6@Gel was studied using the dialysis membrane method. The concentration of C6 in the gels was set as 6 *μ*g/mL. The release medium contained phosphate-buffered saline (PBS; pH 7.4) and Tween-80 (0.5%, w/w) to ensure sink conditions. The shaking rate of free C6 and C6@PM was set at 100 rpm, while the shaking rate of C6@PM@Gel and free C6@Gel were set at 40 rpm. And the temperature was maintained at 37°C. At the scheduled time, 1 mL of the release medium was withdrawn and replaced with an equivalent volume of preheated fresh medium. C6 was quantified using fluorospectro photometer analysis (Shimadzu RF-5301 Spectrofluorophotometer, Japan), with the excitation wavelength set as 458 nm and the emission wavelength set as 497 nm.

#### 2.3.5. In Vitro Retention Behaviors of C6@PM@Gel and Free C6@Gel

The in vitro retention behaviors of the C6@PM@Gel and free C6@Gel were analyzed using near-infrared (NIR) imaging (IVIS Lumina II, PerkinElmer Management, Shanghai). The C6@PM@Gel and free C6@Gel were placed in 48-well plates and shaken at a rate of 40 rpm, with the temperature set at 37°C. NIR imaging of each 48-well plate was performed after 0, 2, 4, and 8 days (Ex = 458 nm, Em = 497 nm).

#### 2.3.6. In Vitro Cytotoxicity Assay

Roswell Park Memorial Institute-1640 (RPMI-1640) containing 10% fetal bovine serum and 1× antibiotic–antimycotic (Gibco, Thermo Fisher Technology, China) was used to culture 4T1 mouse breast cancer cells. The cytotoxicity of various preparations of quercetin against the 4T1 cells was studied using the MTT assay. Briefly, 100 *µ*L of 6 × 10^3^ 4T1 cells in RPMI-1640 complete medium were seeded in the 96-well plate and incubated for 24 hr at 37°C. Next, the medium was removed, and the cells were treated with 100 *µ*L of fresh RPMI-1640 complete medium or medium containing different concentrations of blank micelles, free quercetin, and Qu@PM solution and incubated for up to 24 and 48 hr. The morphology of the 4T1 cells was observed under an inverted microscope (Lecia DM2700M, Leica Microsystems, Shanghai). Subsequently, 100 *μ*L of MTT solution (5 mg/mL in PBS) was added to each well, and the cells were incubated at 37°C for 4 hr. The culture medium was then removed and substituted with dimethyl sulfoxide to dissolve the precipitated formazan. Finally, the absorbance was measured at 490 nm using a microplate reader (ELx800™, BioTek Instruments Inc.) and calculated cell viability using Equation (3).

(3) $$\begin{array}{c} Cell\;viability=\frac{\left(OD_{drug}-OD_{blank}\right)}{\left(OD_{control}-OD_{blank}\right)}. \end{array}$$

#### 2.3.7. Cell Apoptosis Analysis Using Flow Cytometry

Two milliliters of 1 × 10^5^ 4T1 cells in RPMI-1640 complete medium were seeded in a 6-well plate and incubated for 24 hr at 37°C. Next, the medium was removed, and the cells were treated with 2 mL of fresh culture medium or medium containing free quercetin and Qu@PM solution at a final concentration of 20 *μ*g/mL and incubated for up to 24 hr. Then, 5 *μ*L of Annexin V-FITC was added to each sample, and the samples were incubated in the dark for 10 min. Next, 10 *μ*L of PI was added. Each sample was analyzed using a flow cytometer (Navios, Beckman Coulter Commercial Enterprise, China).

#### 2.3.8. Statistical Analysis

All data are expressed as mean ± standard error of the mean. Statistical analysis was performed using SPSS 25.0, and between-group differences were assessed using Student's *t*-test. *p* < 0.05 was considered statistically significant.

## 3. Results and Discussion

### 3.1. Characterization of Qu@PM and Qu@PM@Gel

**Figure 1:**
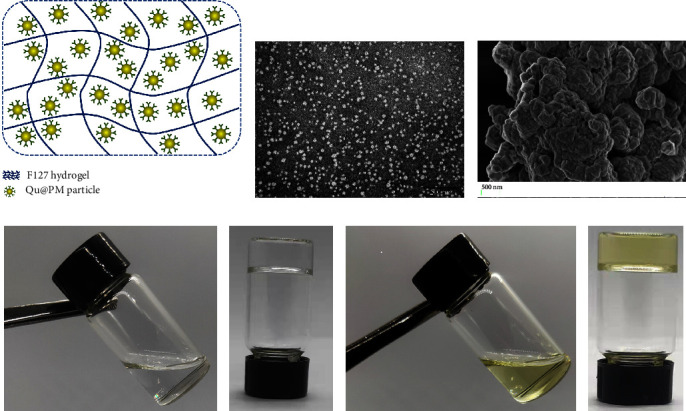
Characterization of the F127 hydrogel drugs. (a) Schematic graph of the Qu@PM@Gel. (b) TEM of the Qu@PM and (c) SEM of the Qu@PM@Gel. (d) Appearance of the blank hydrogel at 25°C and (e) the blank hydrogel at 37°C and (f) the drug-loaded hydrogel at 25°C, and (g) drug-loaded hydrogel at 37°C.

A schematic illustration of quercetin micelles and the quercetin micellar thermosensitive hydrogel is presented in Figure 1(a). Our previous research results have shown that the average particle size of the Qu@PM was approximately 15 nm and that the zeta potential was around −10.31 mV [15]. The EE and DL of Qu@PM were 95.06% and 3.10%, respectively. TEM images showed that Qu@PM was spherical and around 15 nm in diameter (Figure 1(b)). Moreover, because Qu@PM was loaded into F127, SEM showed that Qu@PM@Gel could gather small micellar balls together in a condensed state (Figure 1(c)).

As shown in Figure 1(d)–1(g), both the blank hydrogel and Qu@PM@Gel were uniform and transparent, but the latter eventually became yellowish-green due to the original color of quercetin. Both the gels had a free-flowing consistency at ambient temperatures (Figures 1(d) and 1(f)) and instantly formed solid gels at 37°C (Figures 1(e) and 1(g)).

### 3.2. Rheological Studies

**Figure 2:**
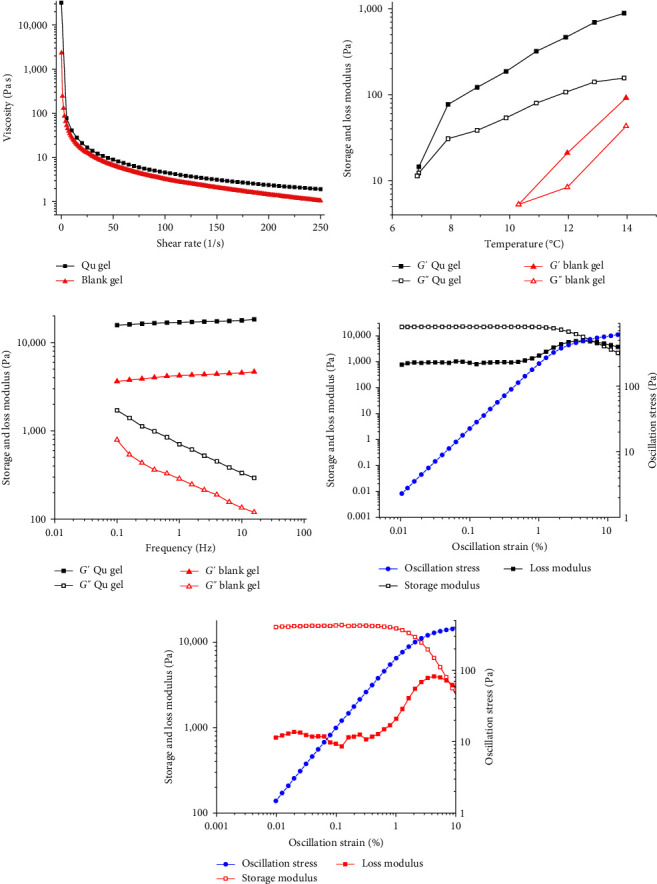
Rheological properties of the different hydrogel systems. (a) Shear-thinning properties of the Qu@PM@Gel and blank hydrogel. (b) The gel formation temperature of the Qu@PM@Gel and blank hydrogel. (c) Frequency sweep of the Qu@PM@Gel and blank hydrogel, with the frequency ranging from 0.01% to 20%. (d) and (e) Linear viscoelastic region of the Qu@PM@Gel hydrogel and blank hydrogel, respectively.

Because thermosensitive hydrogels have the property of shear-thinning, they show good injectability, which is desired for their administration. As shown in Figure 2(a), the viscosity of the blank hydrogel and Qu@PM@Gel decreased with an increase in the shear rate and hold capacity of the hydrogels (*G*′ > *G*″). After adding Qu@PM, the viscosity decreased with an increase in the shear rate, and no difficulty was encountered when injecting these hydrogels through a syringe. This demonstrated the shear-thinning capacity of the hydrogels.

Moreover, the *G*′ and *G*″ values of the two hydrogels at varying temperatures (4–40°C) were measured; the gel formation temperature was considered the temperature at which the liquid turned into an immobile gel when the *G*′ equaled the *G*″ value. As shown in Figure 2(b), the *G*′ value gradually increased with an increase in the temperature. When the *G*′ equaled the *G*″ value, the liquid turned into a semisolid state, which is the critical point, signaling the formation of the hydrogel. The temperatures at which the blank hydrogel and Qu@PM@Gel were formed were 6.9 and 10.3°C, respectively, indicating the fine gel-forming ability of the Qu@PM@Gel.

As illustrated in Figure 2(c), the *G*′ value outweighed the *G*″ value for two samples with increased frequency, and the semisolid gel exhibited excellent gel performance at 37°C. In addition, the hydrogel network was not destroyed when the oscillation ranged from 0 to 100 Hz. As shown in Figures 2(d) and 2(e), when the amplitude ranged from 0% to 10%, the *G*′ value was greater than the *G*″ value for both samples, and the elasticity was dominant. With the strain ranging from 0.01% to 1%, the modulus was not significantly altered, illustrating the linear viscoelastic region of the two samples.

### 3.3. In Vitro Retention of C6@PM@Gel and Free C6@Gel

**Figure 3:**
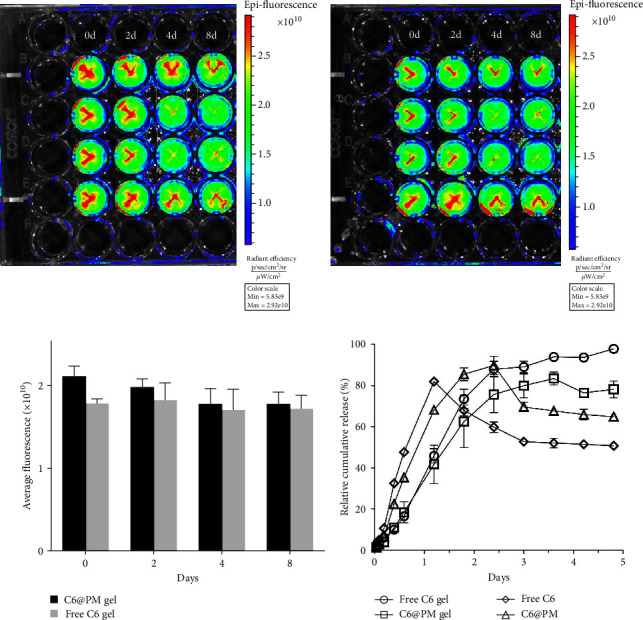
In vitro diffusion study. (a) The C6@PM composite hydrogel. (b) The free C6 hydrogel. (c) Quantitative results of fluorescence intensity. (d) In vitro release of the C6@PM composite hydrogel and free C6 hydrogel.

Using NIR imaging, we could identify the diffusion behaviors of the two drug particles by directly visualizing the C6 signals. As depicted in Figures 3(a) and 3(b), both the hydrogels were diffused, and the fluorescence of C6 attenuated over time. The C6 micellar group diffused slower as compared with the free C6 group (Figure 3(c)). In addition, the drug particles still existed at Day 8 in the hydrogel, highlighting the fine-sustained release of the prepared hydrogel formulation.

### 3.4. In Vitro Release Behaviors of Free C6, C6@PM, C6@PM@Gel, and Free C6@Gel

Figure 3(d) illustrates the in vitro release profiles of free C6, C6@PM, C6@PM@Gel, and free C6@Gel. It was observed that over 80% of C6 was released from free C6 within 24 hr, and the C6@PM released 68% of C6, whereas the C6@PM@Gel and free C6@Gel released only 40% of C6 during the same period of time. Free C6, C6@PM, C6@PM@Gel, and free C6@Gel presented a release profile of first-order kinetics, and the fitting equations were *y* = 0.587 (1−e^−0.117*x*^) (*R*^2^ = 0.9141), *y* = 0.748(1−e^−0.067*x*^) (*R*^2^ = 0.8960), *y* = 0.912(1−e^−0.028*x*^) (*R*^2^ = 0.9620) and *y* = 1.132(1−e^−0.024*x*^) (*R*^2^ = 0.9734) for these four systems, respectively.

### 3.5. Morphological Analysis and the Biocompatibility of Blank Micelles

**Figure 4:**
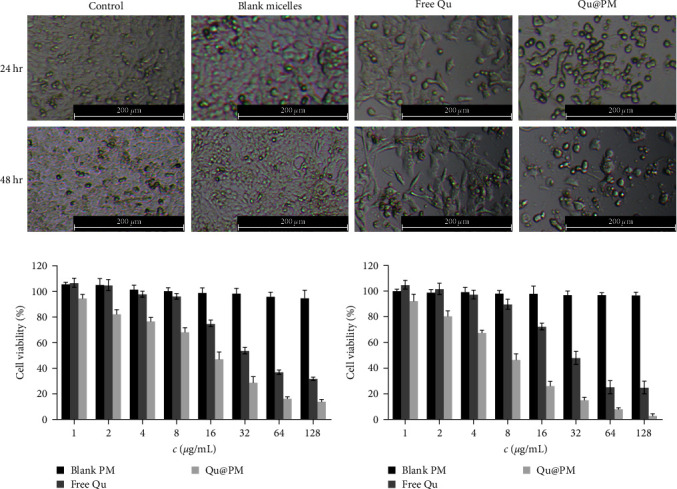
(a) Morphological changes in the 4T1 cells after treatment with various formulations for 24 and 48 hr. Cytotoxicity of the free quercetin and Qu@PM on 4T1 cells after treatment with the indicated drugs for (b) 24 hr and (c) 48 hr.

The morphologies of the 4T1 cells treated with the different drug formulations at a final concentration of 16 *μ*g/mL for 24 and 48 hr were evaluated with varying degrees of cell density decreases (Figure 4). The following logic was used: cell density = control > blank micelles > free quercetin > Qu@PM. The cell density of Qu@PM was obviously decreased under a microscope. Based on the duration of treatment, Qu@PM group could significantly affect the behaviors in cell morphologies, e.g., shrink, shedding, and disruption.

### 3.6. In Vitro Cytotoxicity Assay

**Figure 5:**
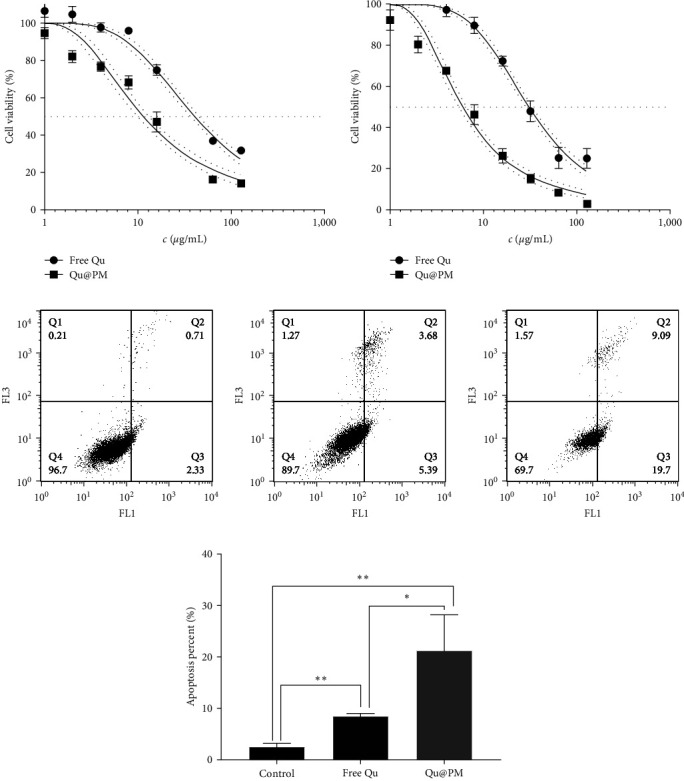
4T1 cell viability curve at (a) 24 hr and (b) 48 hr. (c) Apoptosis evaluation of micelles against 4T1 cells. (d) Quantitative analysis of the flow cytometry results ( ^*∗*^*p* < 0.05,  ^*∗∗*^*p* < 0.01).

The cytotoxicity of the free quercetin and Qu@PM on the 4T1 cells at 24 and 48 hr is shown in Figures 5(a) and 5(b). The inhibition of both drug formulations was dose-dependent for the 4T1 cells. The inhibitory effect of Qu@PM on the 4T1 cells at 24 and 48 hr was elevated in comparison with that of free quercetin. The IC_50_ values of free quercetin were 40.93 and 30.83 *μ*g/mL and of Qu@PM were 11.56 while 6.03 *μ*g/mL at 24 and 48 hr, respectively, indicating that the inhibition of Qu@PM was greater than that of free agents (Figures 5(a) and 5(b)). Qu@PM could significantly enhance the antitumor effects of quercetin because (1) micelles have small-scale dominance that display enhanced performance, such as greater tissue permeability and stronger inhibition to cancer [15]; (2) quercetin core's micelles have sustained release effects, further realizing the endurance effects [15]; and (3) quercetin can inhibit P-glycoprotein efflux and thus reverse the multidrug resistance of tumors [18].

### 3.7. Cell Apoptosis Analysis Using Flow Cytometry

Apoptosis is defined as a type of programed cell death under tight genetic control, and its progress is regulated by different types of genes [19]. As shown in Figures 5(c) and 5(d), we detected the ability of different drug formulations to induce 4T1 cell apoptosis. The percentages of the apoptotic cells in the control group, free quercetin group, and Qu@PM group were 3.04%, 9.07%, and 28.79%, respectively. Compared with the control group, both treatment groups have significant differences, indicating that Qu@PM could play greater roles in the induction of tumor cell apoptosis, the reason for which may be that Qu@PM increases cellular drug uptake or alters the apoptotic pathway [20].

## 4. Conclusion

In this study, we developed and characterized the Qu@PM@Gel, which is a thermosensitive composite hydrogel system. Rheological studies demonstrated that the Qu@PM@Gel could be injected at room temperature and that it formed desirable hydrogels to ensure that the drug is locally released for longer durations at physiological temperatures. The PEG coating of micelles enhances the distribution and stability of drug nanoparticles in the hydrogel system, which have better compatibility with the F127 hydrogel. In vitro release experiments showed that the drug delivery system, whose released time exceeded 96 hr, exhibited good sustained release and good stability, as demonstrated further by the NIR imaging results. The results of the in vitro pharmacodynamics assays showed that treatment with Qu@PM mediated a significantly greater inhibition of 4T1 cells as compared to treatment with free quercetin. It also inhibited cellular activity and enhanced the associated apoptotic effects, suggesting that this drug delivery system has excellent antitumor efficacy. Taken together, the quercetin core's micelles gel system is injectable and can be locally stored with excellent performance, which supports the use of and research on novel drug delivery systems using insoluble flavonoids, including quercetin.

## Data Availability

All the data supporting the findings of this study are available within the paper.
